# Supplementary material for: Genomic Alterations, Gene Expression Profiles and Functional Enrichment of Normal-Karyotype Acute Myeloid Leukaemia Based on Targeted Next-Generation Sequencing
Source: Cancers (Basel). 2023 Feb 22;15(5):1386. doi: 10.3390/cancers15051386 (PMC10000176; doi:10.3390/cancers15051386)
Supplement: Supplementary file 1 [file cancers-15-01386-s001.zip › Supplementary File S1.pdf]

Table SW1. HGC VariantPlex® Myeloid panel

ABL1 ▶	*DCK* ▶	*KIT* ▶	*RPS14* ▶
ANKRD26 ▶	*DDX41* ▶	*KMT2A* ▶	***RUNX1*** ▶ ■
ASXL1 ▶ ■	*DHX15* ▶	*KRAS* ▶	*SF3B1* ▶
ATRX ▶	*DNMT3A* ▶	***LUC7L2*** ▶ ■	*SH2B3* ▶
BCOR ▶ ■ ♣	*ETNK1* ▶	*MAP2K1* ▶	*SLC29A1* ▶
BCORL1 ▶	***ETV6*** ▶ ■	*MPL* ▶	*SMC1A* ▶
BRAF ▶	***EZH2*** ▶ ■	*MYC*	*SMC3* ▶
BTK ▶	*FBXW7* ▶	*MYD88* ▶	*SRSF2* ▶
CALR ▶	***FLT3*** ▶ ■ ♣	***NF1*** ▶ ■	*STAG2* ▶
CBL ▶ ■	*GATA1* ▶	*NOTCH1* ▶	*STAT3* ▶
CBLB ▶	*GATA2* ▶	*NPM1* ▶	***TET2*** ▶ ■
CBLC ▶	*GNAS* ▶	*NRAS* ▶	***TP53*** ▶ ■
CCND2 ▶	*HRAS* ▶	*PDGFRA* ▶	***U2AF1*** ▶
CDC25C ▶ ■	*IDH1* ▶	*PHF6* ▶	*U2AF2* ▶
CDKN2A ▶ ■	*IDH2* ▶	*PPM1D* ▶	***WT1*** ▶ ■
CEBPA ▶	***IKZF1*** ▶ ■	*PTEN* ▶	*XPO1* ▶
CSF3R ▶	*JAK2* ▶	*PTPN11* ▶	***ZRSR2*** ▶ ■
CUX1 ▶ ■	*JAK3* ▶	***RAD21*** ▶ ■	
CXCR4 ▶	***KDM6A*** ▶ ■	*RBBP6* ▶	

DNA obtained from patients during the presentation and after attainment of CR1/CR2 were subjected to Archer® HGC VariantPlex® Myeloid panel targeted DNA sequencing that covered 75 hotspot genes (73 single nucleotide variants (SNVs)/indels ▶, 22 copy number variants (CNVs) ■ and two internal tandem duplications (ITDs) ♣.

Supplementary S1. *ASXL1* NM_015338.5:c.1934dup (VAF <5%) Validation

Gene and mutations	Dx1	Dx2	Dx3	Dx4	Dx6	Dx7
ASXL1 NM_015338.5:c.1934dup	Test	Test	Test	Test	Test	Control
Variant allele frequency (%)	3.95	3.32	3.33	3.67	3.52	-

1. Primer Design

Primer	Forward	Reverse	Insertion Information
ASXL1	GGTTAAAGGTCAGCCCACTTA	CAGTAGTTGTGTTCGCTGTAGA	COSM1411076 c.1934dup (Insertion)

2. PCR Reaction Methodology

The PCR master mix used for primer optimisation and reaction was Phusion PCR Master Mix (Thermo). Setup of mastermix as below:

PCR master mix	1X (20ul) optimisation	2X (20ul) Sanger seq
2x Phusion PCR master mix	10	10
Forward Primer (10uM)	1	1
Reverse Primer (10uM)	1	1
nuclease-free water	6	6
DNA (40ng)	2	2

Primer optimisation:

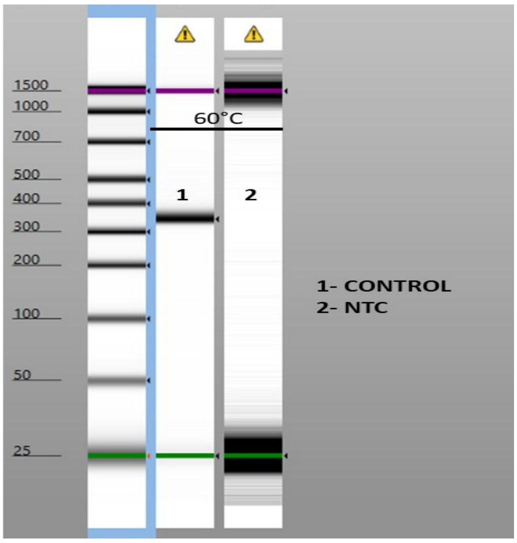

Conclusion: Proceed with 60°C annealing temperature

PCR for Sanger sequencing:

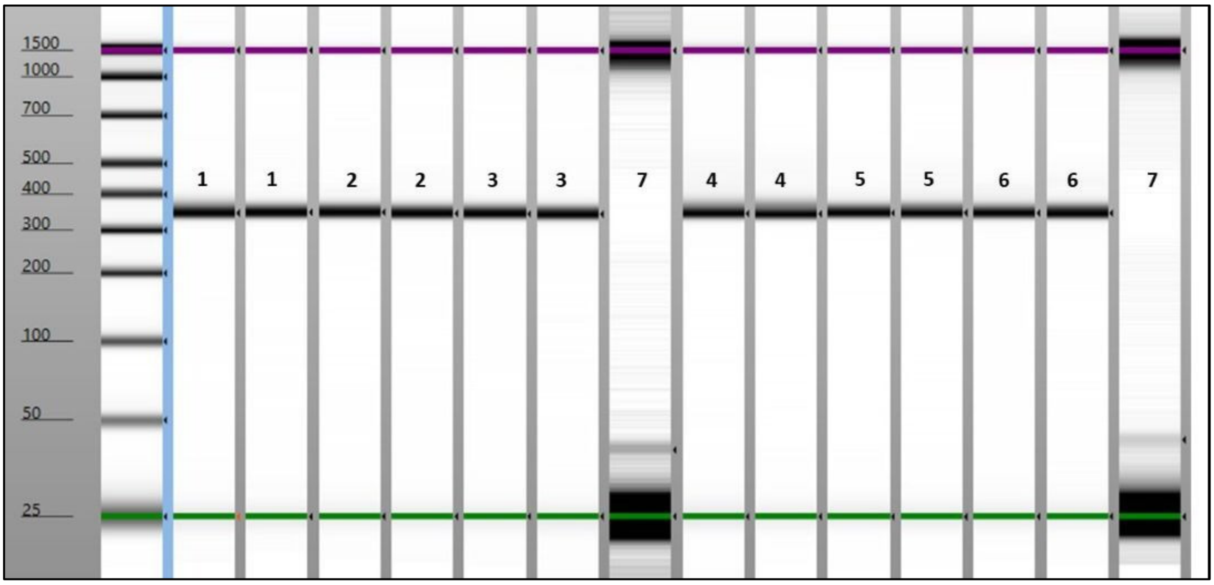

Lane	Sample
1	DX1
2	DX2
3	DX3
4	DX4
5	DX6
6	DX7 (Control)

3. PCR Product Purification for Sanger Sequencing

Kit: Geneall Exspin PCR SV (103-102)
The protocol was as per the manufacturer's recommendation, and proceeded with DNA sequencing.

4. Sanger Sequencing Analysis

ASXL1 **-** c.1934dup (Insertion)

Sample	c.1934dup (Insertion)
DX1	No insertion detected
DX2	No insertion detected
DX3	No insertion detected
DX4	No insertion detected
DX6	No insertion detected
DX7 (Control)	No insertion detected

Results

1. DX7_P control sample

Sanger Seq data (Forward): CACTGCCATCGGAGGGGGGGGTGGCCCGGGTGGAGGTGGCG

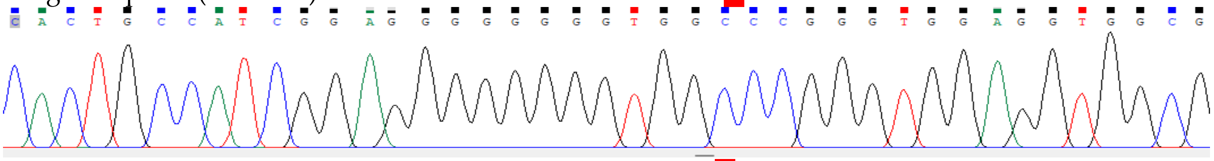

Sanger Seq data (Reverse): CACTGCCATCGGAGGGGGGGGTGGCCCGGGTGGAGGTGGCG

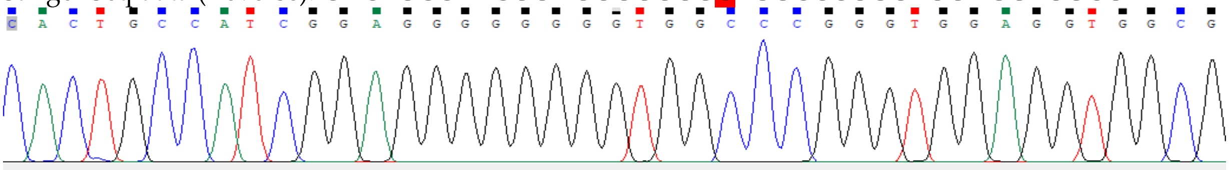

2. DX1_P - No insertion detected

Sanger Seq data (Forward): CACTGCCATCGGAGGGGGGGGTGGCCCGGGTGGAGGTGGCG

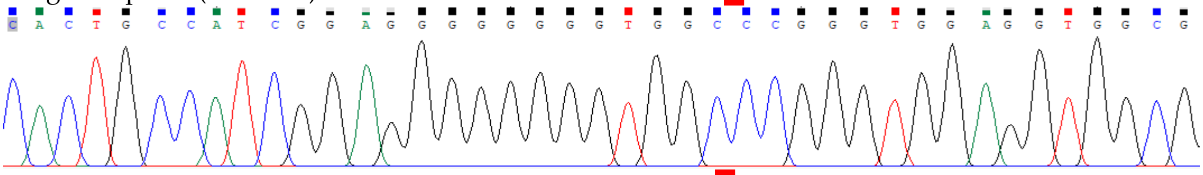

Sanger Seq data (Reverse): CACTGCCATCGGAGGGGGGGGTGGCCCGGGTGGAGGTGGCG

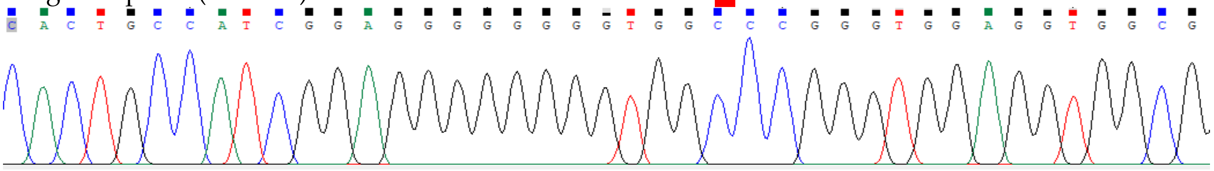

3. DX4_P- No insertion detected

Sanger Seq data (Forward): CACTGCCATCGGAGGGGGGGGTGGCCCGGGTGGAGGTGGCG

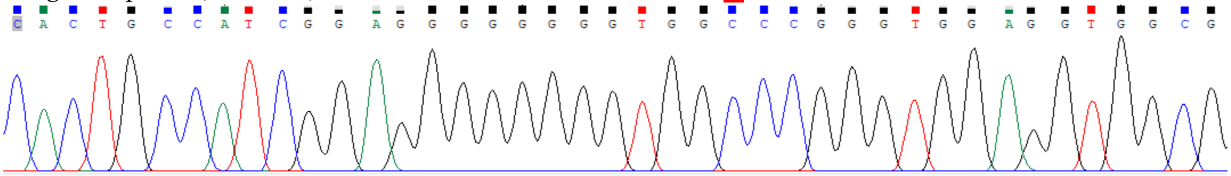

Sanger Seq data (Reverse): CACTGCCATCGGAGGGGGGGGTGGCCCGGGTGGAGGTGGCG

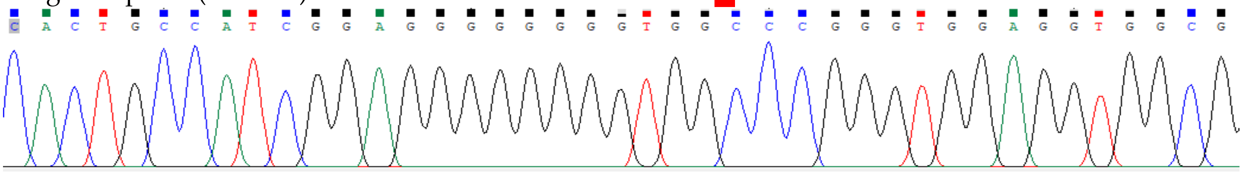

4. **DX6_P- No insertion detected**

Sanger Seq data (Forward): CACTGCCATCGGAGGGGGGGGTGGCCCGGGTGGAGGTGGCG

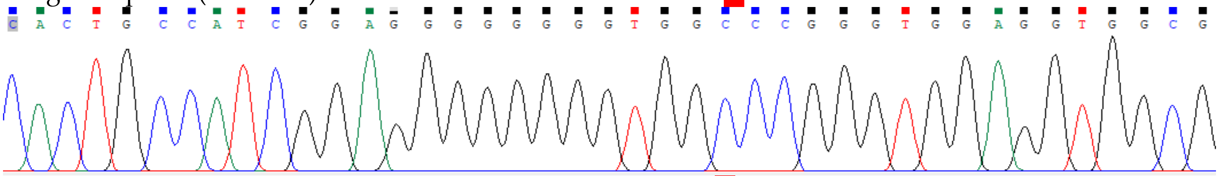

Sanger Seq data (Reverse): CACTGCCATCGGAGGGGGGGGTGGCCCGGGTGGAGGTGGCG

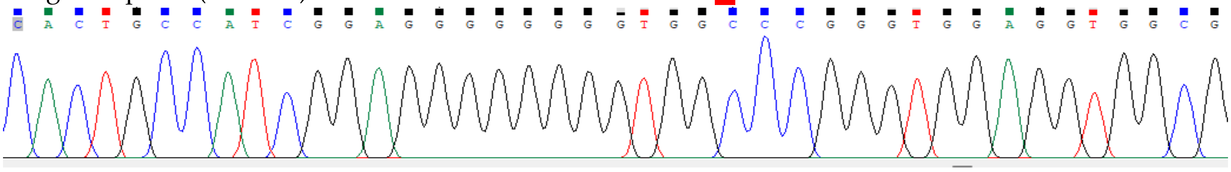

5. **DX3_P - No insertion detected**

Sanger Seq data (Forward): CACTGCCATCGGAGGGGGGGGTGGCCCGGGTGGAGGTGGCG

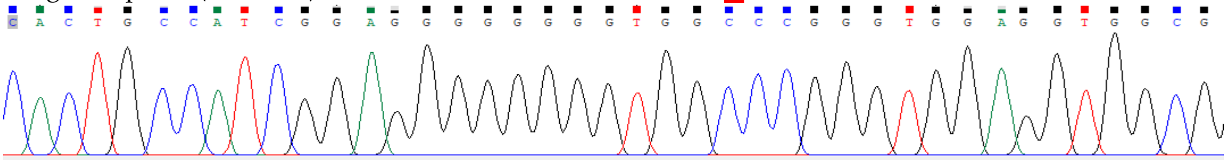

Sanger Seq data (Reverse): CACTGCCATCGGAGGGGGGGGTGGCCCGGGTGGAGGTGGCG

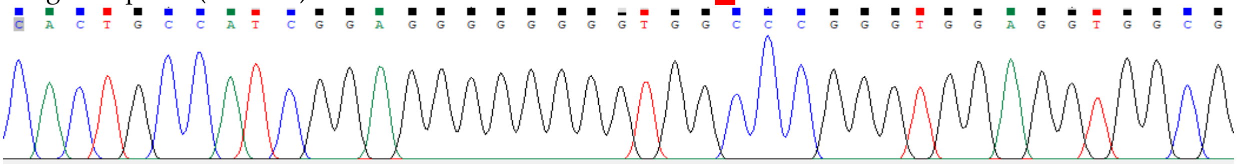

6. **DX2_P - No insertion detected**

Sanger Seq data (Forward): CACTGCCATCGGAGGGGGGGGTGGCCCGGGTGGAGGTGGCG

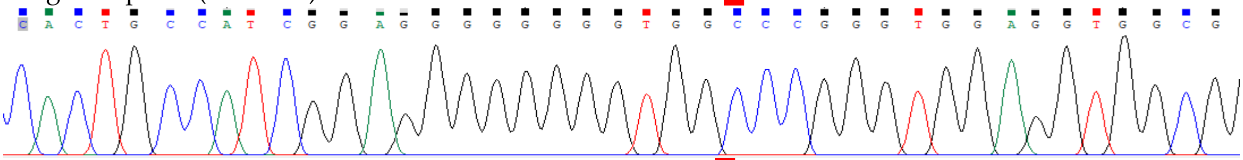

Sanger Seq data (Reverse): CACTGCCATCGGAGGGGGGGGTGGCCCGGGTGGAGGTGGCG

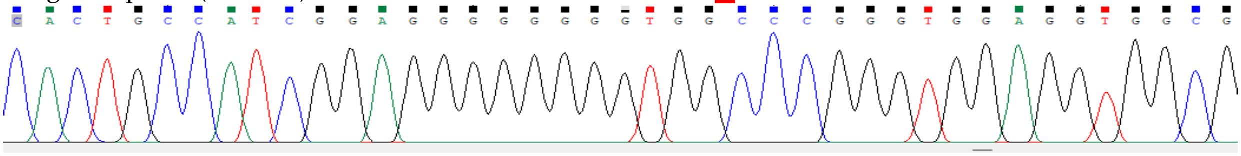

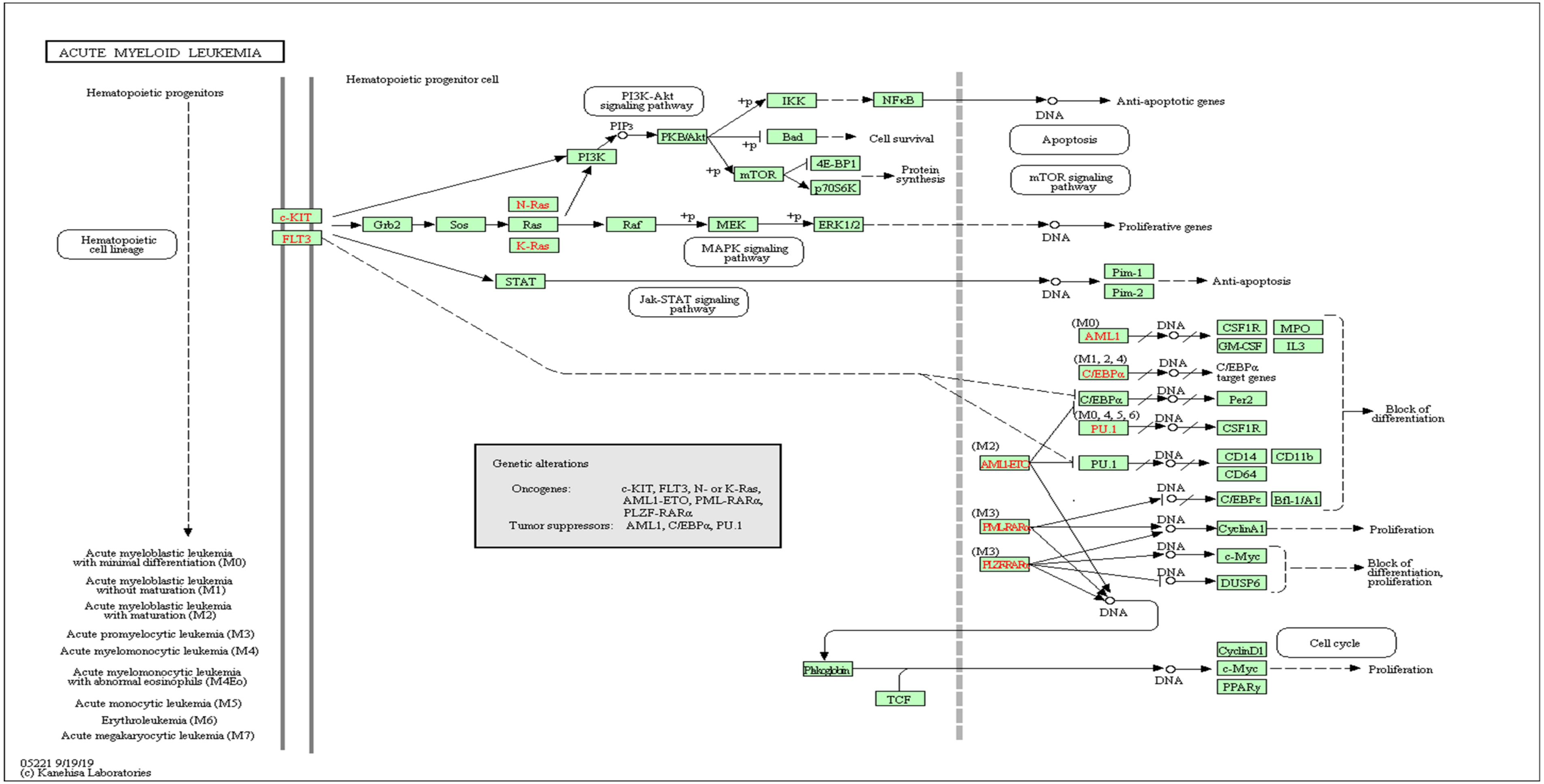

Figure SW1. Acute myeloid leukaemia pathway. Genes that are affected are highlighted in red in the diagram. As shown, genes that are located upstream of this pathway were involved, including *FLT3, HRAS* and *RUNX1* in selected patients (Dx 1-8), as shown in Table 1, and Figure 4. (Image from KEGG resource)

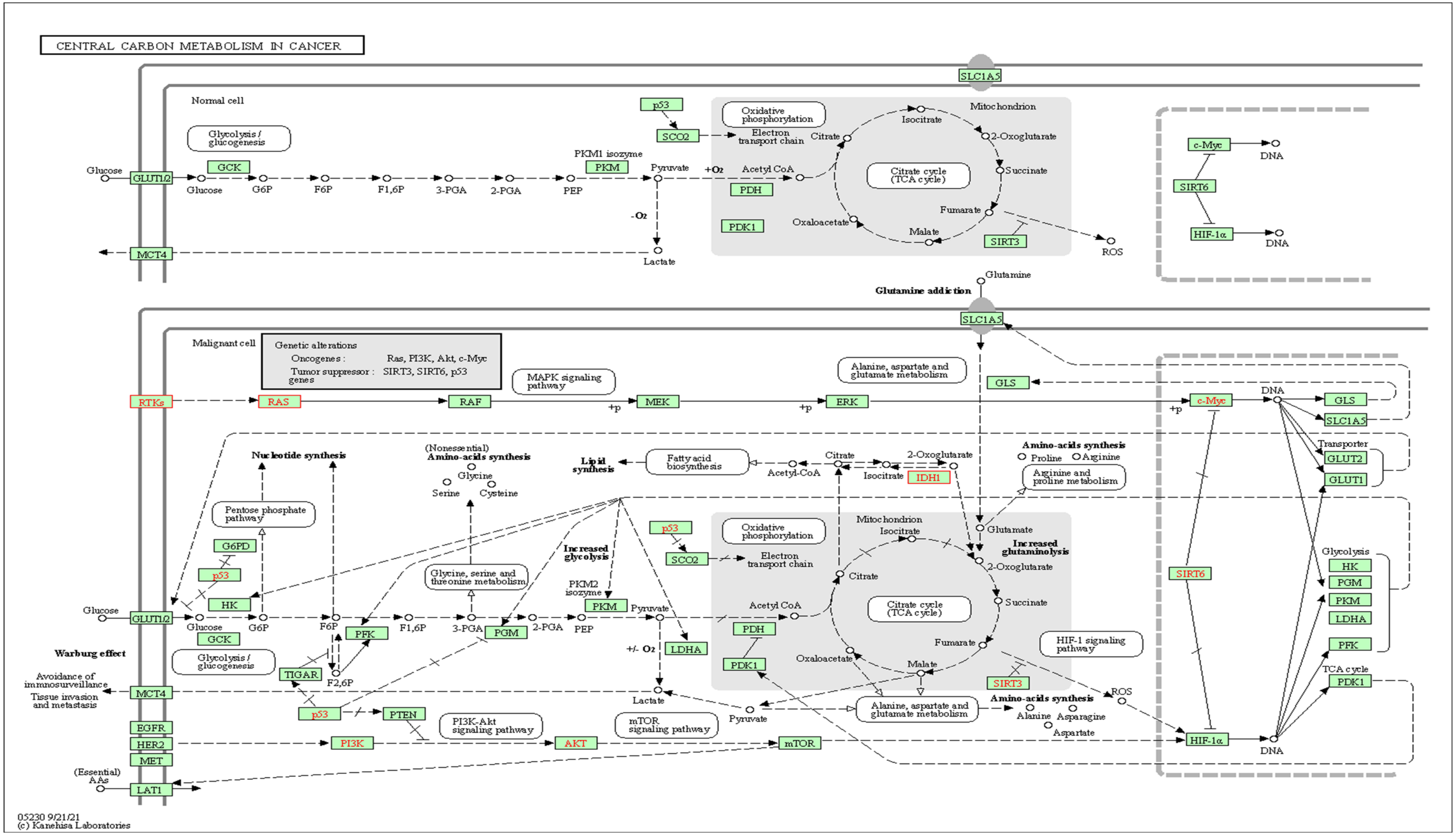

Figure SW2. Central carbon metabolism in cancer. Genes that are affected are highlighted in red in the diagram. The *HRAS* (RAS family gene) is located upstream of the MAPK signalling pathway, and several other genes, including *IDH1*, are located downstream of carbon metabolism processes. (Image from KEGG resource)

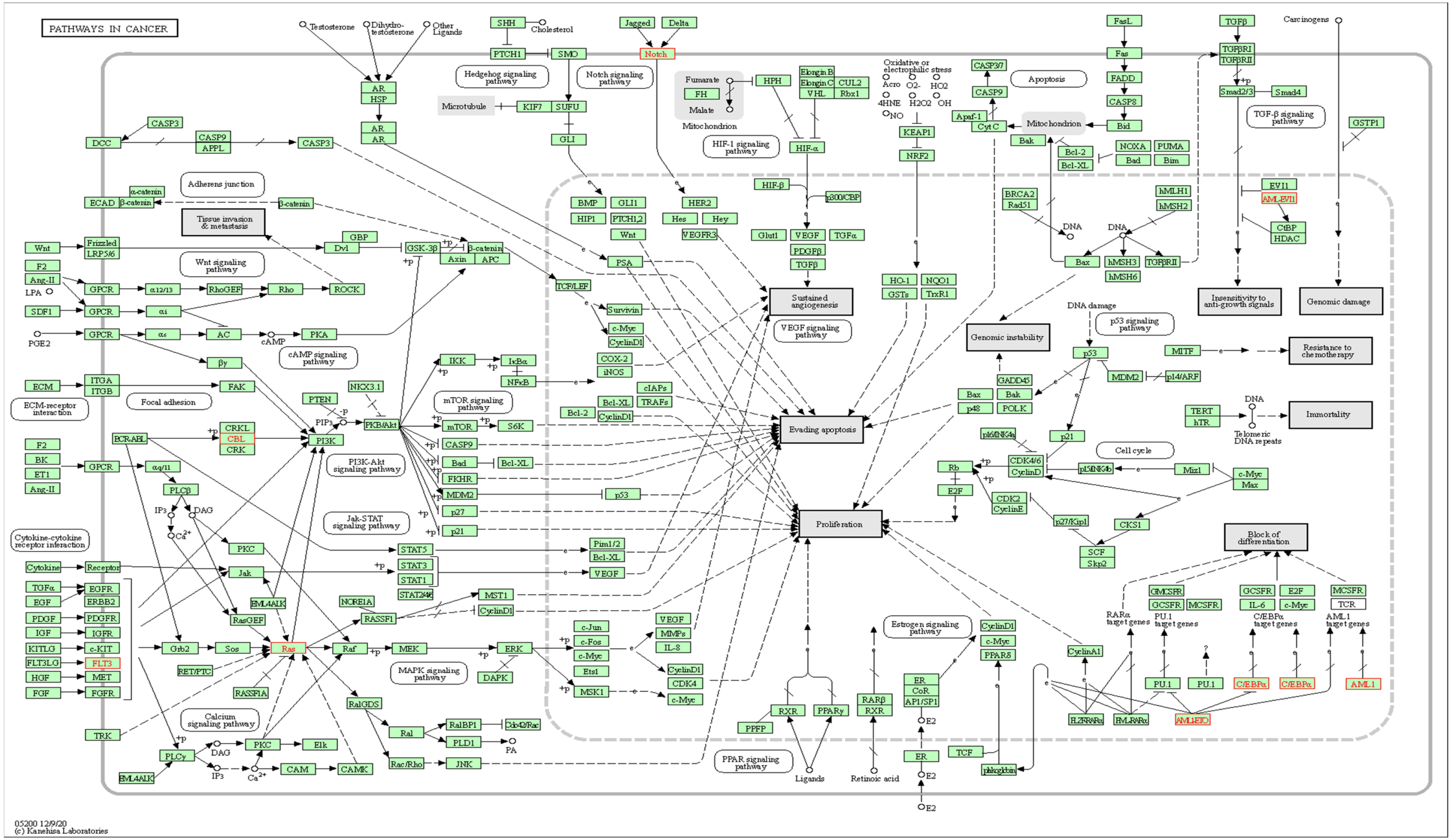

Figure SW3. Pathways in Cancer. Genes that are affected are highlighted in red in the diagram. Several hub genes at the intersection of various pathways were involved, including *FLT3* (known hub-gene), *HRAS* and *RUNX1*, which significantly impact the leukaemogenesis of AML-NK in this study. (Image from KEGG resource)

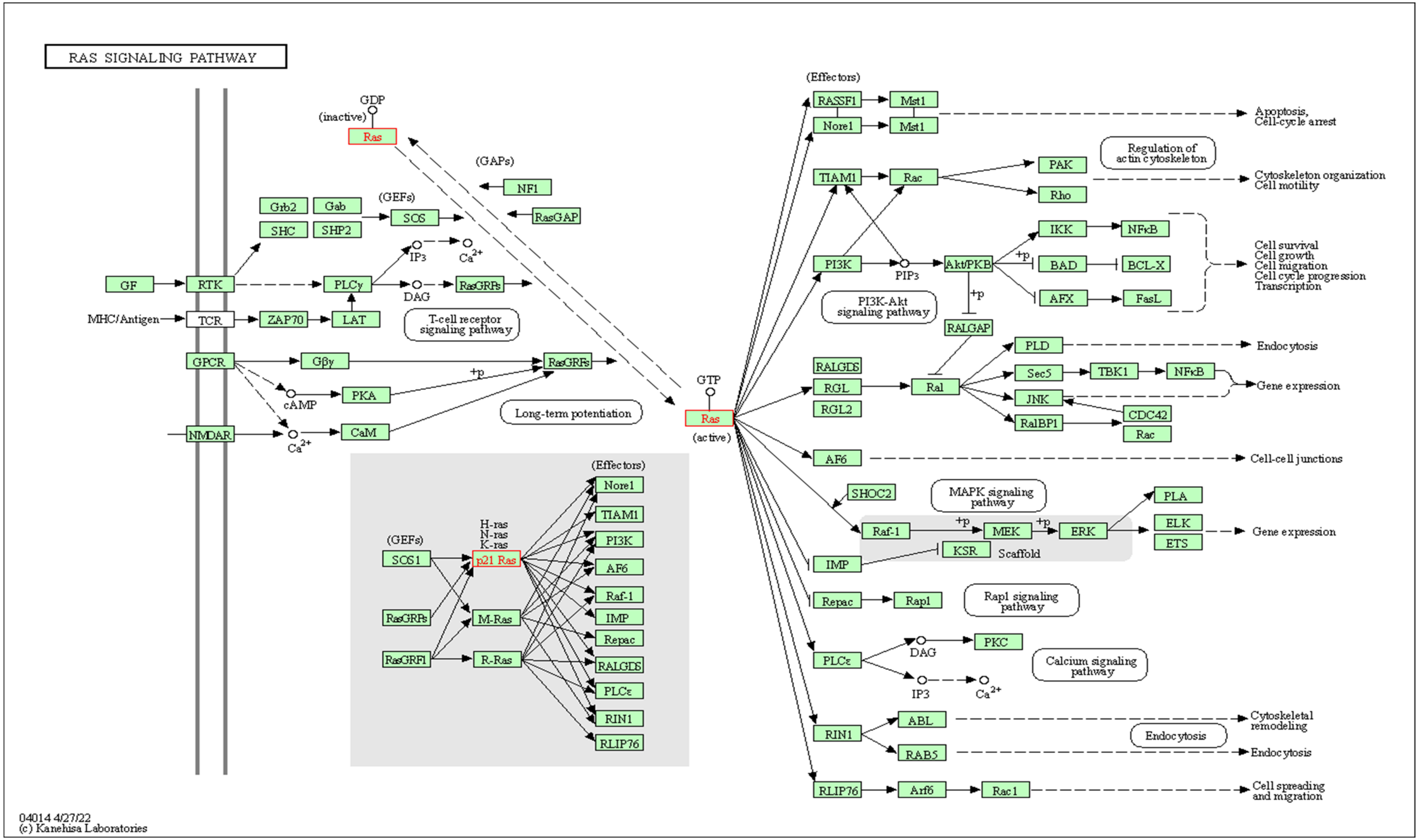

Figure SW4. RAS signalling pathway. *HRAS* gene (RAS gene family) is a critical hub gene involved in several signalling pathways, as shown in this diagram. (Image from KEGG resource)

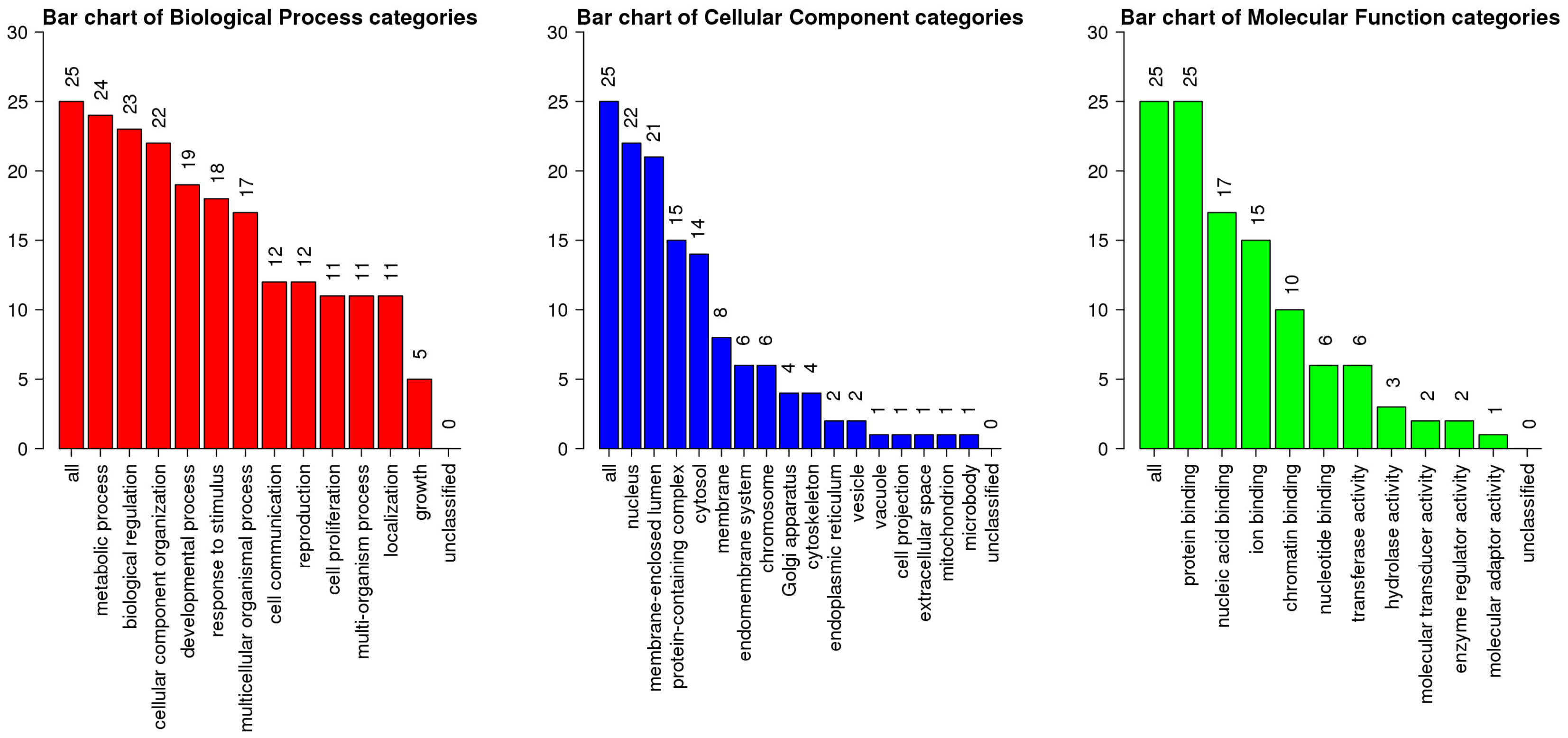

Figure SW5. Gene Ontology (GO) summary for the 26 genes with somatic variants

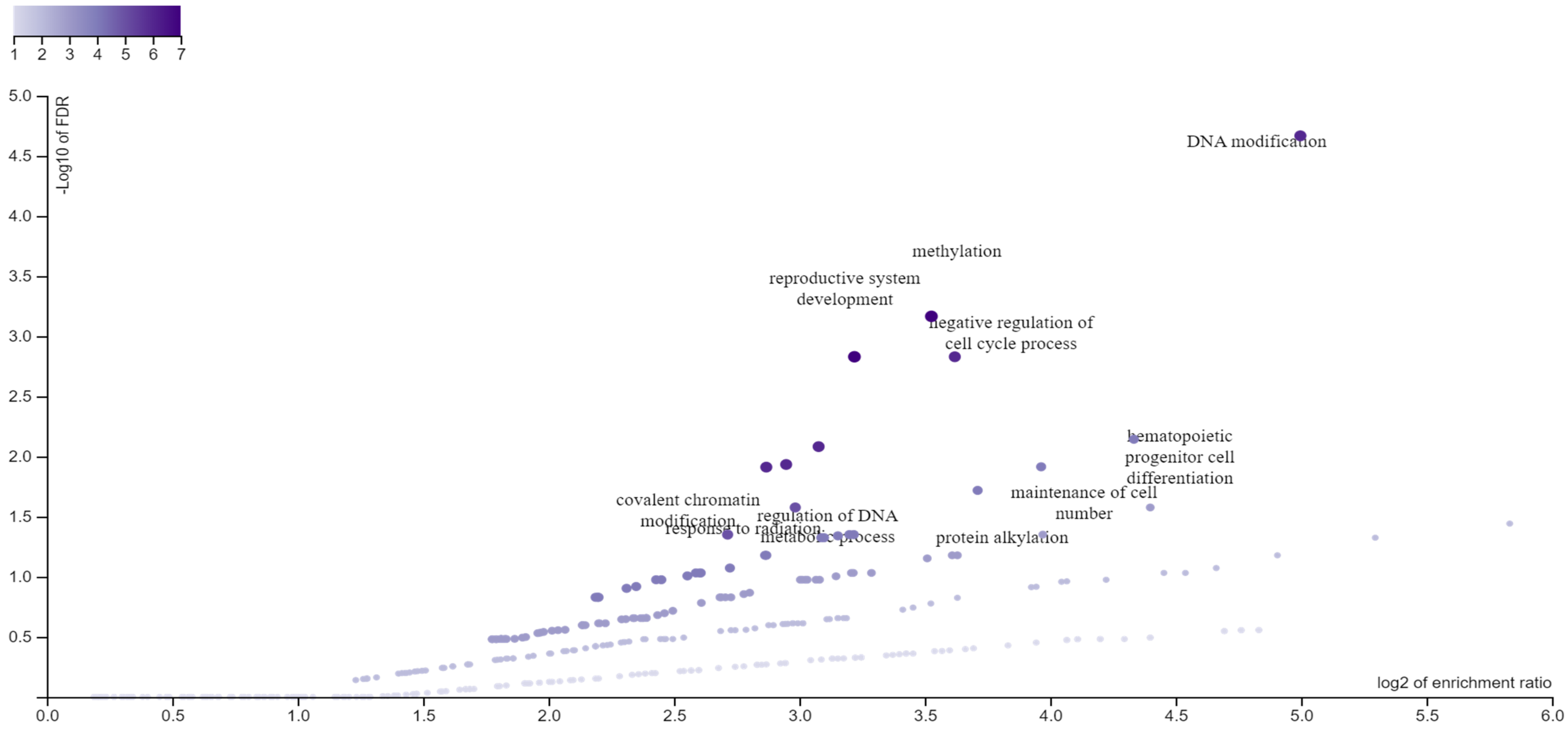

Figure SW6. Volcano plot depicting Biological Process GO for the 26 genes with somatic variants

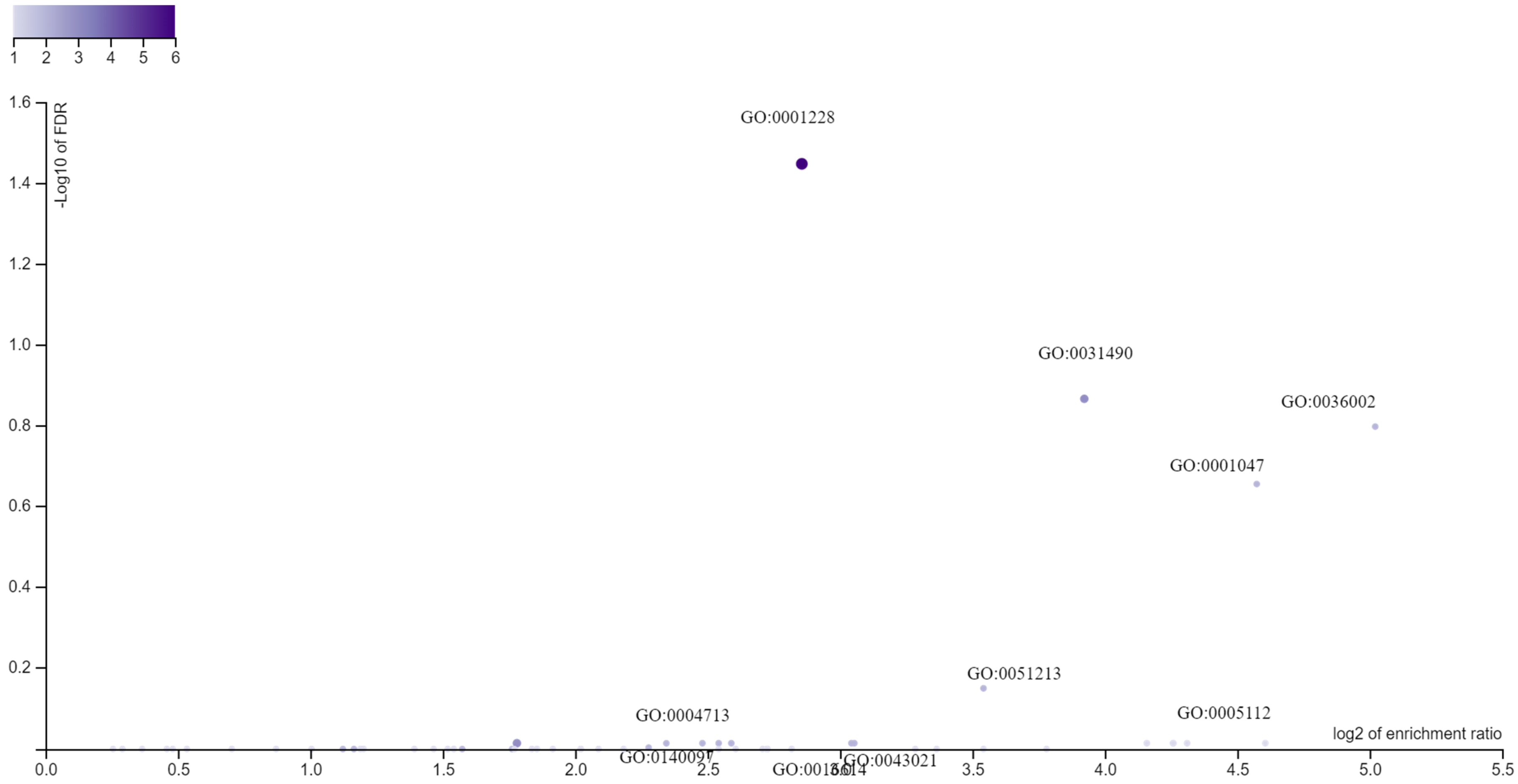

Figure SW7. Volcano plot depicting Molecular Function GO for the 26 genes with somatic variants

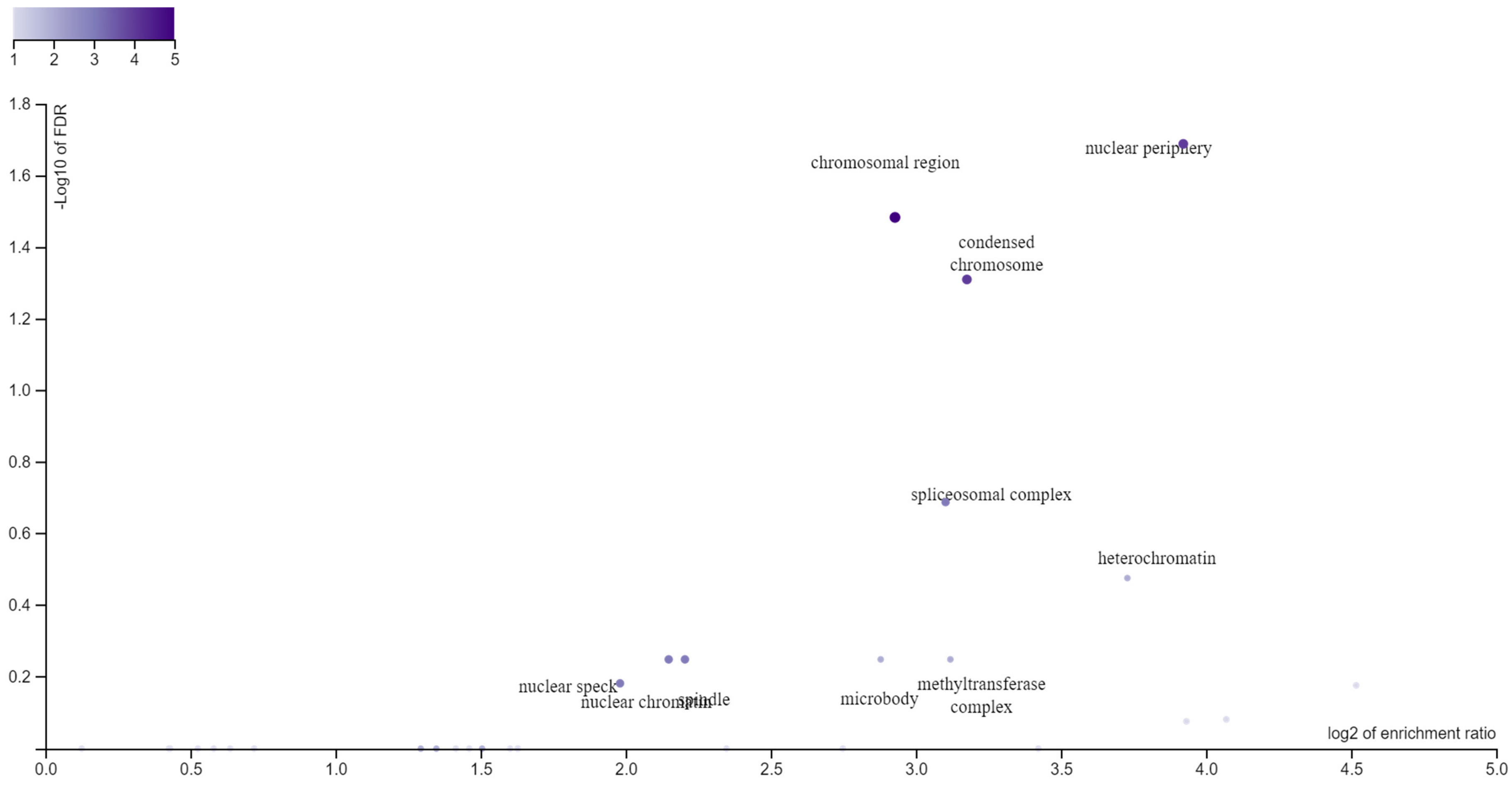

Figure SW8. Volcano plot depicting Cellular Component GO for the 26 genes with somatic variants

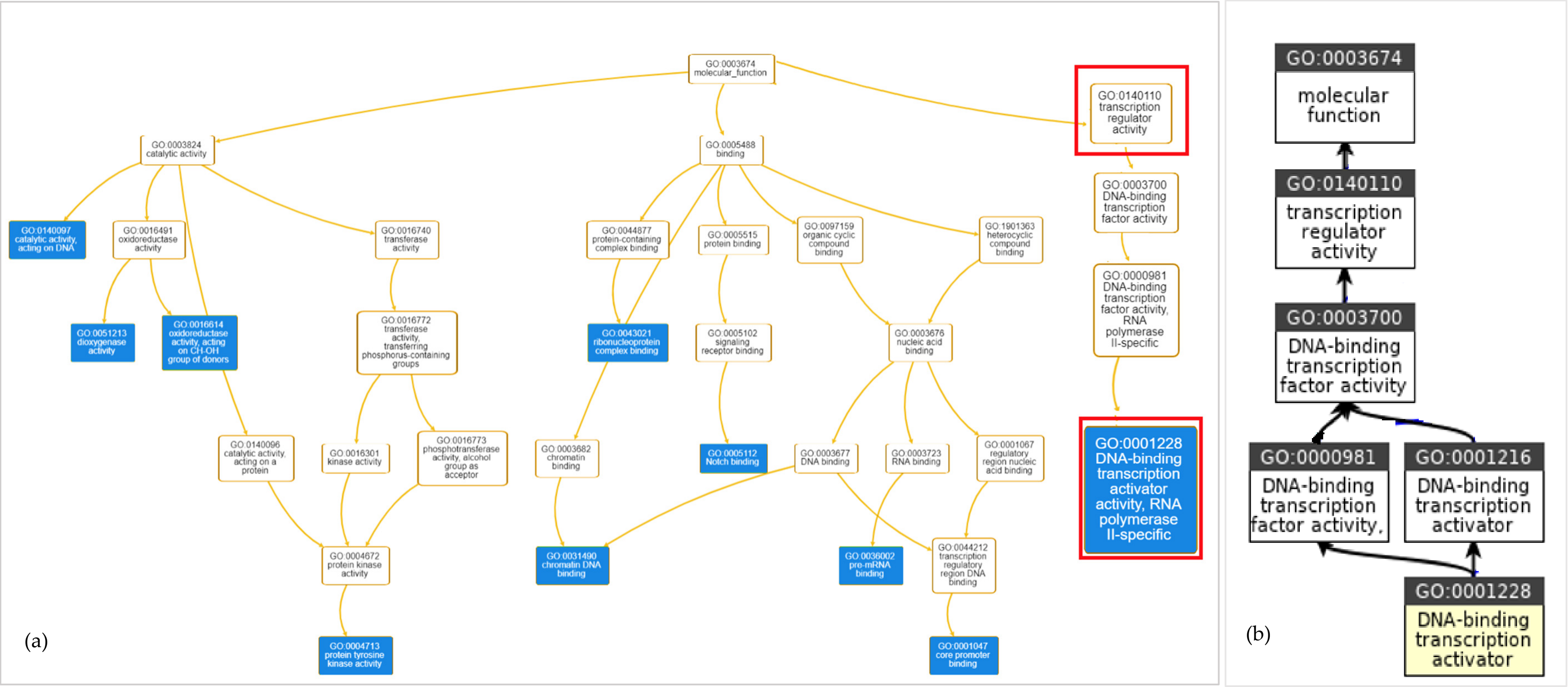

Figure SW9. Directed acyclic graph (DAG) (a) and Quickgo graph of the GO for MF enrichment of 26 genes somatic variants and. The branches depict the containment relationships and blue boxes indicates GO terms that are significantly over-represented in this cohort. The most the most enriched GO MF category was DNA-binding transcription activator activity, RNA polymerase II-specific (GO:0001228) ($p<0.01$, FDR=0.4) as highlighted in red boxes along with ancestral GO term GO:0140110 (Transcription regulation activity).
